# Annotations

**Published:** 1896-02-22

**Authors:** 


### ANNOTATIONS.

### A Novel Cure for Colds.

A GOOD many new cures for colds have lately been published. Perhaps the most novel and the most hopeful is Dr. Schnee's. Schnee employs what may be considered a massage variant. He percusses the terminal branches of the nerves supplying the mucous membrane of the nose with a small hammer made of india-rubber. Slight shocks upon terminal nerves have the effect, as has been experimentally demonstrated, of contracting the blood vessels. That is, they excite the activity of the vaso-motor nerves. Stronger shocks produce dilatation of the same blood vessels, no doubt, by over stimulating and so exhausting the vaso-motors. Here, then, we have a method of exercising a great deal of control over those nasal blood vessels, whose altered condition constitutes the initial stage of coryza. In the inception period of a cold, what is wanted is to set up contraction of nasal and naso-pharyngo-laryngeal blood vessels. For this purpose slight "tappings" with the india-rubber hammer are to be resorted to. The locality to which the percussion should be applied is the forehead, just above the root of the nose; and the "taps" should follow a line extending horizontally outwards over the eyebrows. The "tapping" should be frequently interrupted and resumed, since it is manifest that continuous "tapping" would over-stimulate and finally exhaust the vaso-motors, thus exaggerating the very evil the remedy is designed to cure. In cases of chronic catarrh the "tapping" is also valuable, only in this condition, it must be of a heavier degree and more sustained; what is wanted being first a free secretion of mucus, and afterwards a return to a condition of normal vascularity. The method is interesting, and based on physiological reasoning. Let us hope it will prove as effective in practice as it sounds scientific in descridtion.

### A Danger to the Medical Profession.

THE *British Medical Journal* of February 8th contains the details of a proposal for altering the articles of association of the British Medical Association in such a manner as to make it possible for that body to add to its other functions the work of medical defence, and on the same date a leading article refers with approval to this communication as being "an unanswerable document." Now, the point we wish to draw attention to is this. According to the original memorandum, the main purposes of the Association are the promotion of medical science and "the maintenance of the honour and interests of the medical profession," not, be it observed, of the members of the Association alone. In the draft resolution drawn up by their lawyer the same phraseology is used, the Association being allowed to grant sums of money out of their funds: (1) For the protection of the interests of the medical profession, (2) for the protection and defence of individual members of the medical profession, &c., not a word being said about any preference for the members of the Association. But when we come to the details of the provisional scheme proposed by a committee of members, we find that the first duty entrusted to the proposed Board is "The defence of individual members attacked by persons not members of the Association," and nowhere is any suggestion made to defend any individual member of the profession unless at the same time a member of the Association. This is a somewhat serious matter, for elsewhere it is stated that this same committee recognised that the plan of subsidising private defence societies would not be satisfactory, and it is impossible not to see that if this scheme should really take rcot it might interfere greatly with existing associations which at present are open to all respectable members of the profession. It certainly would be an odd result of all the agitation which has gone on for so long in favour of some organised plan of self-defence among the members of the medical profession, if it were to eventuate in such defence being unattainable except by subscribing to a particular medical association. In our view the proposals of this committee should not be accepted without some modification in regard to all medical practitioners, as they threaten a danger to the medical profession.

### The Increase of English Suicides.

WAS it Burke who said that "the price of liberty is eternal vigilance"? If we had a physiological Burke who was also a patriot he would tell us that the price of a high standard of national health is an intelligent obedience to physiological teachings. The fifty-seventh annual report of the Registrar-General of Births, Marriages, and Deaths has just been published. The report is for the year 1894. It contains many significant facts. One of the most significant of them all is this, that there were registered during the year 2,729 deaths from suicide, a rate of 91 per million of the population, and the highest suicide rate on record. There must be a reason for this, or reasons. We ought to want to know what the reasons are; and we ought to want to make suicide less common, not more common. If many of us are indifferent on this subject it augurs ill for the future of our country. Poverty does not seem to have been the main cause of the increase because the country in 1894 was relatively well off. Nor, apparently, was general ill-health the cause, because the death-rate was not the highest, but almost the lowest on record during the whole year. The low death-rate was indeed remarkable, being no more than 16·6 per 1,000, as contrasted with a rate for previous years of 19·2. The birth-rate was, however, low, and that perhaps throws a little side-light upon one of the causes of the increased suicide-rate. A low birth-rate means a low marriage-rate; that is to say, it indicates that fewer people than usual are willing to take upon themselves the burdens and responsibilities of family life. The burdens and responsibilities of family life were probably the main reason why so many persons found it impossible any longer to maintain this mortal conflict. Can we diminish the weight of these burdens as civilisation increases in complexity? It is to be feared we cannot. What, then, can we do? Only one thing—and that is to breed, and rear, and train a hardier population, a population made of sterner stuff. Is this possible to us? It is possible; and a rational physiology, with unerring finger, steadfastly points out the way.

